# Clinical Remarks on Cases of Congenital Syphilis and Rickets1Seen in the Children’s Department of the Syracuse Free Dispensary, October 15, 1892: before the third-year class of the College of Medicine, Syracuse University.

**Published:** 1893-02

**Authors:** A. Clifford Mercer

**Affiliations:** 324 Montgomery Street


### CLINICAL REMARKS ON CASES OF CONGENITAL SYPHILIS AND RICKETS.[1]

By A. CLIFFORD MERCER, M. D.

Of the two cases of scabies you saw in the dispensary a week ago, I shall say nothing beyond what was said when the cases were before you. Of the case of spinal curvature, I shall say only a few words presently. These cases belong to classes of disease which will be fully considered in the departments of dermatology and surgery, respectively.

1. Seen in the Children's Department of the Syracuse Free Dispensary, October 15, 1892: before the third-year class of the College of Medicine, Syracuse University.

I ask you to recall a puny infant of two months, lying in its father's arms, and evidently, from its general appearance, severely ill. You observed its marked anemia, with a slightly ashy tint in its face. You saw in the diaper, as it was removed, a semi-fluid green stool, containing lumps of curdled milk—irregular, white cheesy masses, about the size of peas. On the buttocks, perineum, and about the pudenda you saw a uniformly raw and irritable-looking purplish redness which at its margin on the thighs broke up into more or less discrete small papules. You saw faint remaining stains of a more extended eruption which, I told you, was previously present down the legs. The small papules of the more extended eruption were very symmetrically distributed; on the right calf and heel they were confluent and formed patches corresponding in situation with similar patches on the left calf and heel. Each of you felt of one of the several places in the lateral occipital regions where there seemed to be a hole in the cranium, the bone being replaced by a stiff parchment-like membrane which yielded to gentle pressure and suddenly sprang back as the pressure of your finger was removed, a phenomenon resembling that which sometimes occurs in a grosser way when you press in sharply the bottom of a tin pail. The condition, you were told, was that known as craniotabes, a word meaning cranial wasting: a condition generally believed to be a pressure atrophy due to compression of the soft cranial bones between the developing convolutions of the brain on the one side and the pillow or nurse's arm on the other. This is not a perfectly satisfactory belief; for in some cases the situation of the atrophy would necessitate the presence of some other external pressure, or that the infant rest standing on its head (Dr. A. F. Voelcker, London). You perhaps noticed that during all your handling the infant was but little disturbed, a common indication of severe illness in infants. You did not detect any other sign of disease by your direct examination. From the mother we learned the rash appeared about a week after birth and that the infant had been badly fed on bottle food. From the father and mother, questioned each alone, we could get no history of syphilis in themselves or in their other children. Nevertheless, the case had been recognized from the first as one of congenital syphilis. At the time you saw the case, the diagnosis was confirmed by the improvement which had come as a result of mercurial inunction.

The chief interest in the case lies in the fact that the history got from the parents was of so little value that the diagnosis had to be made on signs, independently of any history. The eruption alone was sufficiently characteristic to establish a diagnosis. The anemia with an ashy tint and .craniotabes are met with in other diseases. The anemia might have been due to the indigestion and chronic diarrhea of which we got a history and of the existence of which we saw evidence in the diaper. Craniotabes for nearly forty years after its definite recognition by Elsässer in 1843 was regarded as a lesion peculiar to rickets. Toward the close of that period, the investigations of M. Parrot[1] in one direction and those of Drs. Lees and Barlow[2] and Dr. Carpenter[3] in another direction showed syphilis to be a large factor in the causation of craniotabes in rickety infants and that syphilis alone caused the lesion in infants not rickety. Further, craniotabes, but much less marked, has been found in apparently healthy young infants. At present then *marked* craniotabes may be regarded as a sign of either syphilis or rickets ; but the more marked the craniotabes, the more surely should you consider the possibility of the presence of con genital syphilis, whether the infant be rickety or not. Our case was young to have rickets, and we detected no sign peculiar to rickets. On the other hand, the infant's age—two months—is one at which congenital syphilis is particularly likely to show itself Infantile syphilis is more common before the fifth month ; rickets is more common after the fifth month. In fact, ordinary rickets is rare before the fifth month. The craniotabes in our case was exceptionally well marked and, especially as there were no signs peculiar to rickets present, was a sign of congenital syphilis. The anemia, the ashy tint in the face, the general appearance of serious illness, as well as the infant's age, were in harmony with the diagnosis of congenital syphilis.

Family histories in cases of congenital syphilis are often negative for one reason or another. Mothers who are not syphilitic may give birth to infants who inherit the taint from the father. Under such circumstances the mother who usually brings the infant to the dispensary and knows nothing about her husband's taint gives a clean history. However, in our case we had an opportunity to interview the father, but with a result as negative as

1. Maladies des enfants. La syphilis héréditaire et la rachitis. Paris, 1886. Transactions of the Pathological Society of London, Vol. xxx., 1879, p. 339.

2. Transactions of the Pathological Society of London, Vol. xxx., 1879, p. 332 ; Vol. xxxi., 1880, p. 236 ; Vol. xxxii , 1881, p. 323.

3. St. Thomas Hospital (London) Reports, Vol. xix., 1889, p, 235.

that got from the mother. The case illustrates the possibility, as well as the necessity which occasionally occurs, of arriving at a diagnosis in cases of infantile disease with little or no aid from histories.

This can only be done by means of close observation, careful thought and large experience, if it is to be done successfully. Enthusiasm, opportunity, and industry sometimes develop ability in objective diagnosis to an almost marvelous degree. To try to observe closely and fully, to interpret the subjective meaning of an infant's behavior — for the infant feels and communicates its feelings to those who understand infantile modes of expression — and to develop or recognize and interpret physical signs of disease in infants and children are particularly the objects of our work together. You will soon note that in developing or recognizing such physical signs the same means are used with which you are acquainted in your clinical work among adults, but that these means usually have to be more carefully and delicately employed and the results are frequently somewhat different and require a somewhat different interpretation. Therefore, you see, there are excellent reasons — a necessity, in fact — for giving some special attention to clinical study of pediatrics. We can, at least, have an ambition to be able to recognize the presence of many of the diseases of infancy and childhood. This ability can only be acquired by practice afforded in some such work as we have in hand. We can have a further ambition to be able to detect the presence of many of the diseases of infancy without asking a question. The consideration of all you see, hear, feel, and smell may be so methodical, full and effective that the answers to your questions will appear to be only items of detail in the broad and pretty accurate idea of the disease you are able to get independently of questions. Such ability has been acquired and is a possibility for which each of you may strive.

However, you will be continually asking questions and using the answers you get to aid you in arriving at a diagnosis. Frequently you will find such answers of great value; at other times, of no value. You will find a part of our work to be learning what questions to ask, how to ask them, and what weight to give the answers as evidence in diagnosis. The recording of the history of the present illness and of the family and its hygiene — so far as they relate to the case — is a matter of routine in hospital work and is usually completed as fully as possible before the direct examination is begun. Sometimes the fullest record and most careful direct examination will make you little wiser about the nature of the case than you were before; but at such times you may have for your consolation knowledge of the fact that even men of exceptional experience and ability from time to time meet with cases which baffle them.

Let us now recall the next patient, a fairly well nourished infant of eighteen months. We watched it sitting on its mother's lap until we saw its head droop—as if the muscles of the neck were too tired to hold the head erect. On throwing off its clothing, you saw the shoulders thrown forward and drooping and the spine rounded to give the trunk as tired a look as that presented by the drooping head. You saw the enlarged wrists and felt the enlargements at the junction of the ribs with the costal cartilages, the so-called "beads" or "rosary." Your attention was called to the open fontanelle. You noted the presence of only ten teeth, the anemia, the flabby muscles, the proportionately enormous globular abdomen. Some of you felt with what ease the femur and tibia slipped about on one another, giving you an idea of the looseness of the infant's joints. The mother told you the infant had walked, but was too weak to do so when she brought it before you. The mother gave us a history of wretched feeding, the infant having been fed anything the family had and at any time of day or night, a history of diarrhea and a history of whooping with a whoop not quite like that she heard when her other children had whooping cough. We did not hear the cough, but you were told the so-called whoop was probably the crowing of laryngismus stridulus. You noticed the infant was inclined to be happy and play. A few weeks previously it took no interest in anything and was still weaker. The infant had improved since it had been fed regularly on a diet limited to plenty of milk, soft poached eggs, bread and gravy, simple puddings and an emulsion of cod liver oil and beef peptonoids.

You were asked to write on slips of paper the name of the disease before you. Three-fourths of the class, to my surprise, handed in slips on which the word rickets was written. I knew you had not had diadetic lectures on pediatrics, and that you were attending your first clinic in pediatrics. It appeared later that your diagnosis was the result of the practical application of your theoretical knowledge of rickets acquired in your pathological work.

You have now seen clinically, as well as pathologically, that rickets is not simply a disease of bones. It is a general disease. You have had evidence in your first case that it involves bone-forming cartilage, ligaments, muscles and other tissues. The affection of bone-forming cartilage was seen in the enlarged wrists and the beaded costo-chondral unions. The lax ligaments and flabby muscles permitted the drooping of the head and trunk and the looseness of the joints generally. The abdominal walls were so weak as to give before the pressure of gas in the alimentary canal, in turn due to decomposing food and functional disease of the stomach and intestine. The anemia was a sign of abnormal blood. The laryngismus stridulus was a spasm sign, one of several which occur in rickets as a result of a functional derangement of the nervous system.

The case presented some of the notable signs of rickets; others which were not present, or not detected, you will see in other cases. For instance, when we come to investigate the case of lateral and rotary spinal curvature in a somewhat stunted-appearing girl of four years, whom you saw for a few moments only, I think we shall find the scoliosis to be a deformity due to rickets of an earlier year; and we may find little remaining of other signs. You will be disappointed if you expect many cases to present to you the full picture you read about. One case will present an apparently accidental selection of signs from the full picture, and another case another selection. You will meet with a variety of selections, or groupings of signs, in different cases of the same disease; and you will, probably, meet with many cases before you will see all the phenomena of that disease, and perhaps you will never see all of them. You will learn that clinical realities are not exactly like book pictures. Only such clinical realities, however, are the objects of our study, rather than the full pictures drawn from the accumulated records of medical literature. The latter will be presented to you in a course of lectures on Pediatrics by Prof. Plant whose lectures, I may say by the way, have long been regarded as among the best given in the Syracuse school. Our work is but supplemental to that course.

Both cases upon which I have briefly dwelt in these fragmentary remarks illustrate the principal thought I wish you to take away with you; and that thought is that it is possible to arrive at a diagnosis in some cases of disease in infants without asking a question. Our questions in the case of congenital syphilis got us answers of negative value, and we were obliged to arrive at a diagnosis without aid from those answers ; and, although our questions got us useful information regarding the case of rickets, we could have arrived at a diagnosis without such information — without, in fact, asking a question.

324 MONTGOMERY STREET.

